# Perceived environment and physical activity: a meta-analysis of selected environmental characteristics

**DOI:** 10.1186/1479-5868-2-11

**Published:** 2005-09-05

**Authors:** Mitch J Duncan, John C Spence, W Kerry Mummery

**Affiliations:** 1 School of Health & Human Performance, Central Queensland University, Rockhampton, Australia; 2 Faculty of Physical Education and Recreation, University of Alberta, Edmonton, Canada

## Abstract

**Background:**

Several narrative reviews have been conducted on the literature examining environmental correlates of physical activity (PA). To date these reviews have been unable to provide definitive summaries of observed associations. This study utilizes meta-analytical techniques to calculate summaries of associations between selected environmental characteristics and PA.

**Methods:**

Published studies were identified from electronic databases and searches of personal files. Studies were examined to determine the environmental constructs most frequently studied. Included studies (N = 16) examined at least one identified construct and determined associations between perceived environmental constructs and PA using logistic regression. Data were analyzed separately for crude and adjusted ORs using general-variance based fixed effect models.

**Results:**

No significant associations emerged between environmental characteristics and PA using crude OR. The perceived presence of PA facilities (OR 1.20, 95% 1.06–1.34), sidewalks (OR 1.23, 95% 1.13–1.32), shops and services (OR 1.30, 95% 1.14–1.46) and perceiving traffic not to be a problem (OR 1.22, 95% 1.08–1.37) were positively associated with activity using adjusted ORs. Variance in PA accounted for by significant associations ranged from 4% (heavy traffic not a problem) to 7% (presence of shops and services).

**Conclusion:**

Results of the meta-analysis support the relevance of perceived environmental characteristics for understanding population PA. These results should encourage the use of comprehensive ecological models that incorporate variables beyond basic demographic information.

## Introduction

The burden of disease attributable to physical inactivity is estimated at $377 million in Australia [1], $2.1 billion in Canada [2] and $24 billion in the U.S. [3], and continues to rise as large majorities of populations remain insufficiently active for health benefits. Research relating to the determinants of physical activity and inactivity has previously focused on determinants at the individual level, largely neglecting physical environments as influences of PA [4]. It is now acknowledged that environments that people build and inhabit provide potential opportunities and barriers to engaging in physically active lifestyles [5,6]. Furthermore, suburban sprawl and the way neighborhoods are designed are related to the physical health and bodyweight status of adults residing in those neighborhoods [7,8]. Thus, research understanding how elements of the natural and built environment influence PA is increasing [9-11].

Findings of existing primary studies and narrative reviews studying the associations between the perceived environment and PA are ambiguous. For example, pathways in close proximity to the home have been found to have a positive association with PA in some instances [12] while showing no association with PA in others [13]. Contrary to intuitive belief, positive associations have been found between the presence of unattended dogs and PA using both subjective [14] and objective [15] measures. Perceptions of neighborhood crime have been found to have both a negative association [15,16] and a lack of association [14] with PA. Similarly, the self-reported presence of street lighting has produced both positive [17] and no association [12,14] with activity using self-report measures and objectively defined geographic information system (GIS) measures [15]. Recent narrative reviews [11,18,19], while providing a useful summary of existing research, have been unable to resolve the aforementioned ambiguities.

Meta-analyses permit the strength and direction of associations between dependent and independent variables to be detailed [20,21]. In comparison to narrative reviews, meta-analyses have predefined inclusion criteria, consider sample size of the study when combining effects, and provide summaries of effects across different outcome measures [22]. In addition, narrative reviews may be subject to inclusion bias, as only studies supporting the reviewer's hypothesis may be included [23-25]. However, meta-analysis is not without its critics. For instance, it has been argued that combining effects from observational studies accentuates the methodological and statistical inaccuracies of included studies [26]. However, the application of appropriate statistical techniques and well defined inclusion criteria can help alleviate some of these concerns [27]. The purpose of this meta-analytic review is to identify the strength and direction of relationships between characteristics of the perceived environment and PA.

## Methods

### Search Procedures

Studies were located from five sources. First, searches of the electronic databases MEDLINE, Proquest, and Infotrac were conducted for the period from 1989 to February 2005. Search terms included, but not limited to, *physical activity*, *exercise*, *walking*, *environment*, *built environment*, and *perceived environment*. Second, a manual search was performed, for the same time period, on the following Journal titles: *Medicine and Science in Sports and Exercise*, *Preventive Medicine*, *American Journal of Public Health*, *American Journal of Health Promotion*, and *American Journal of Preventive Medicine*. These titles were included for manual searches as the majority of studies identified from electronic searches were published in these titles. Third, to identify previously unidentified studies, reference lists from narrative reviews on the topic [11,18,19] were examined. Fourth, a search of each author's personal files was conducted. Finally, a request was sent to an electronic PA list-serve (University of South Carolina Prevention Research Center – USCPRC) soliciting any articles accepted for publication and currently in press.

### Study Inclusion Criteria

Studies were initially included in the meta-analysis if they were published in English and assessed any characteristic of the physical environment in relation to PA. Preliminary searches and coding revealed 50 published studies on the topic assessing 138 separate objectively and subjectively-measured environmental attributes. Each attribute was assessed in a minimum of 1 and a maximum of 15 studies. Because the majority of studies used measures of the perceived environment, the current review included only those constructs assessed using measures of the perceived environment. Finally, studies went through a three-stage inclusion process: 1) Environmental constructs represented by a minimum of at least five effect sizes (ES) were included in the meta-analysis (i.e., presence of PA facilities; presence of sidewalks, shops, and services; high levels of crime; street lighting; unattended dogs in the neighborhood); 2) Studies were included only if they assessed the association between the perceived environment and PA using logistic regression and reported sufficient details for ES calculation (i.e., Odds Ratios [OR] and 95% Confidence Intervals [CI]); and, 3) in cases where a series of papers reported on populations that were not independent of one another [14,28] only a single report that satisfied all inclusion criteria from the series was included in the analysis [14].

### Data Extraction

All data were extracted independently by the first author. Where associations between characteristics of the physical environment and PA were reported by separate ethnic groups [14,29], or gender [30-32], the ORs were entered as separate cases. Included environmental constructs were coded separately within the analysis. In addition to OR and 95% CI for each case, several variables were coded for descriptive purposes, including characteristics of the sample (sample size, gender, ethnicity, level of sample [local, state, national], geographic location of sample [urban, rural, mixed], and methodology [theoretical basis, PA measure, mode of PA, reliability and validity of PA and environment measures]). The frequency of these descriptive characteristics are summarized by environmental characteristic in Table 1. Study Design (cross-sectional, experimental, longitudinal) were also coded, however, all studies satisfying inclusion criteria were cross-sectional. When studies reported variables as tertiles, the middle tertile was not included in the analysis in order to conserve the dichotomous nature of the environmental constructs [33-35]. In some instances the referent category used in the initial analyses was opposite to that used in the majority of other cases for the construct (i.e. "Yes" reference category when majority of studies used "No"). When this occurred (n = 5), such cases were removed from the calculation of the ES. While it is possible to reverse categories, the original fourfold table is required to recalculate the OR with the reversed referent category [36]. However these original fourfold tables were not reported in any of the excluded manuscripts.

**Table 1:** Contribution of the Coded Variable to Total Number of ESs Summarized Environmental Characteristics

	Environmental Characteristic							
Coded Variable	PA Facilities	Sidewalks	Shops & Services in Walking Dist.	Heavy Traffic is Present	High Crime is Present	Street Lighting is Present	Unattended Dogs are a Problem	Total

**Gender**								
Men	6.67	-	9.09	6.25	-	-	-	3.03
Women	66.67	68.75	81.82	68.75	69.23	86.67	69.23	72.73
Mixed	26.67	31.25	9.09	25.00	30.77	13.33	30.77	24.24
								
**Ethnicity**								
Combined	40.00	31.25	27.27	37.50	30.77	13.33	30.77	30.30
Nat. Amer.	6.67	12.50	9.09	6.25	7.69	13.33	7.69	9.09
Caucasian	6.67	12.50	9.09	6.25	7.69	13.33	7.69	9.09
African Amer.	26.67	25.00	27.27	25.00	30.77	33.33	30.77	28.28
Latino	20.00	18.75	27.27	25.00	23.08	26.67	23.08	23.23
								
**Country of Sample**								
US	86.67	93.75	90.91	87.50	100.00	100.00	100.00	93.94
Australia	-	6.25	9.09	12.50	-	-	-	4.04
England	13.33	-	-	-	-	-	-	2.02
								
**Geographic Location of Sample**								
Urban	26.67	25.00	81.82	25.00	30.77	26.67	30.77	33.33
Rural	13.33	12.50	18.18	12.50	15.38	13.33	15.38	14.14
Mixed	60.00	62.50	-	62.50	53.38	60.00	53.85	52.53
								
**Theoretical Basis of Study**								
Ecological	60.00	75.00	54.55	75.00	69.23	86.67	69.23	70.71
SCT	-	-	18.18	-	-	-	-	2.02
NS	40.00	25.00	27.27	25.00	30.77	13.33	30.77	27.27
								
**PA Mode Assessed**								
Walking	13.33	-	18.18	-	-	-	-	4.04
Trans. Walking	-	6.25	9.09	-	-	-	-	2.02
Sufficient LTPA	13.33	37.50	-	12.50	15.38	26.67	15.38	18.18
Sufficient PA	73.33	56.25	72.73	87.50	84.62	73.33	84.62	75.76
								
**PA Measure**								
BRFSS	13.33	12.50	-	13.33	15.38	-	15.38	10.20
BRFSS + NPA	13.33	37.50	-	13.33	15.38	40.00	15.38	20.41
SEID	-	6.25	9.09	-	-	-	-	2.04
S-IPAQ	-	-	-	13.33	-	-	-	2.04
7D-PAR	-	-	18.18	-	-	-	-	2.04
NS	13.33	-	-	-	-	-	-	2.04
WPA	60.00	43.75	72.73	60.00	69.23	60.00	69.23	61.22

Notes: Consult studies included in current analysis for citations of PA Measures used.

Percentages are reported as the percentage of effect sizes derived from coded variables in each environmental category.

NS – Not Specified

SEID – Study on Environmental and Individual Determinants of Physical Activity Measure

WPA – Women and Physical Activity Measure; NHI – National Health Interview

S-IPAQ – Short Form International Physical Activity Questionnaire

Because several studies report associations between environmental variables and PA using adjusted ORs, separate analyses were conducted for unadjusted and adjusted ORs. In the case of adjusted ORs, we coded for the variables that were adjusted for in the logistic regression.

### Statistical Analysis

To stabilize the variance of OR and to adjust for the nature of OR (where values of 0.5 and 2 hold equivalent strengths in opposite directions), ORs were transformed to their natural logarithms (ln). The resultant lnOR are approximately normally distributed with positive and negative values representing positive and negative associations respectively [37]. Variance surrounding each OR were calculated using the following formula

              Variance OR_i _= [ln(*OR*_*u*_/*OR*_*i*_)/1.96]^2^

Where OR_u _is the upper confidence interval of the _i_th OR, and OR_i _is the OR of the _i_th study. Confidence intervals and the crude OR from each case were used to estimate the overall OR for each environment variable using a general variance-based fixed effect model [38].

              OR_s _= *e*∑[(*w*_*i *_× ln *OR*_*i*_)]/∑*w*_*i*_

Where OR_s _is the summary OR for the specified variable, w_i _is the weight of the _i_th study, which is the inverse of _i_th study's variance and ln OR_i _is the natural log of the OR for the _i_th study [38]. Homogeneity of variance was tested across studies by the Mantel-Haenszel method using the following formula:

              *Q *= ∑[*w*_*i *_× (ln *OR*_*mh *_- ln *OR*_*i*_)^2^]

Where W_i _is the weight of the _i_th study, and OR_mh _is the natural log of the summary OR for the dependent variable. The Q statistic provides a test of whether the group of effect sizes represents a common population effect. A significant Q statistic indicates the effect does not represent the population of studies and that a search for moderator variables is warranted. Variance in PA explained by each variable (R^2^) was calculated using the procedure proposed by Nagelkerke [39].

## Results

### Descriptive Summary of Sample Studies

Table 1 presents a summary of the coded characteristics that may influence relationships between perceptions of the environment and PA. Across all environmental categories, most of the included studies were conducted in the USA, two studies were conducted in Australia and one study was conducted in the U.K. Native Americans (n = 9) were the least studied ethnic population within the sample, and African Americans (n = 28) were the most studied ethnic population. Most effect sizes (n = 72) were drawn from female populations whereas few effect sizes (n = 3) were drawn from exclusively male populations. The majority of study populations were drawn from local geographic regions. Most studies utilized ecological models of health behavior; however, two studies [40,31] did not specify an underlying theoretical basis. Physical activity outcome variables were comprised largely of people meeting sufficient physical activity criteria [41], including sufficient leisure-time physical activity (n = 18) [14] and sufficient physical activity (n = 75) [42]. The prominent PA assessment method was the Women's Physical Activity Survey (n = 60) used in the Women's Cardiovascular Health Network Project [43]. This was the prominent PA assessment method as a large number of included studies were part of the Women's Cardiovascular Health Network Project.

### Unadjusted Associations (Crude Odds Ratios)

Using crude ORs, no variables demonstrated a significant association with PA (Table 2).

PA facilities was the only variable to exhibit significant heterogeneity among effect sizes (Q[6] = 64.41, p < 0.05). Unfortunately, the limited number of effect sizes within PA facilities precludes any search for moderators to resolve this heterogeneity.

### Adjusted Associations

Age, income and education level were the most commonly adjusted for variables in the original studies. When individually adjusted ORs were combined, four significant associations emerged. People who reported the presence of PA facilities were more likely to engage in PA than those not reporting proximal infrastructure (OR = 1.20, 95% CI. 1.06–1.34). Similarly, people reporting the presence of sidewalks, compared to those reporting the absence of sidewalks, were more likely to be physically active (OR = 1.29, 95% CI. 1.17–1.41). Those people reporting the presence of shops and services within the neighborhood were more likely to engage in activity compared to people not reporting their presence (OR = 1.30, 95% CI. 1.14–1.46). People reporting that heavy traffic was not a problem were more likely to engage in PA compared to those reporting heavy traffic was a problem (OR = 1.22, 95% CI 1.08–1.37). Examination of Q statistics revealed that PA facilities Q(7) = 22.58, p < 0.05, and the presence of shops and services Q(6) = 25.22, p < 0.05, both displayed significant heterogeneity of variance. Once again, the limited number of effect sizes within these two variables prevents any search for moderators to resolve this heterogeneity.

Individual constructs displaying significant associations with PA were further analysed using Nagelkerke's R to determine the amount of variance accounted for in PA. The presence of heavy traffic explained the least amount of variance (R^2 ^= 0.04), PA facilities and sidewalks explained 5% and 6% of physical activity variance respectively, whilst shops and services explained the greatest amount, accounting for 7%.

## Discussion

The purpose of this review was to quantitatively summarize the associations between selected perceived environment variables and PA from individual studies. Our findings confirm previous suggestions that the perceived environment has a modest, yet significant association with PA [54,55]. This is evidenced by individual variables explaining relatively small amounts of variance (4–7%) in PA; however, the contribution of these potential changes to community behavior may be great. Since people living in a particular environment can be influenced by that setting [55], favorable alterations to communities may produce small changes in behaviors of entire populations [54]. Therefore, identifying and modifying environments to produce positive changes in PA are important. Individual level interventions promoting initial changes in PA can be complimented by interventions creating activity friendly environments, to assist in maintaining positive changes in PA. Therefore, multilevel interventions targeting individuals and communities are likely to be the most effective in changing PA.

Reporting the presence of proximal PA facilities, sidewalks, shops and services and that traffic was not a problem were all positively associated with PA. Although previous narrative reviews [11,19] were unable to provide consensus concerning how perceptions of traffic influence PA, the current review found positive associations across studies between the absence of traffic and PA using adjusted ORs. Since it has been noted that many road systems are designed without the needs of pedestrians in mind [56], it is time for planners to recognize the health relevance of their work. For instance, providing sidewalks separated from roads, pedestrian refuge islands, increased street lighting and roundabouts are all highly effective methods of reducing pedestrian crashes [56]. Engineering modifications can reduce traffic volumes and speeds if implemented effectively [57]. Although the potential for road modifications to reduce traffic volumes and increase individual PA may be small in magnitude, such modifications may contribute to sustainable positive changes in PA levels across the community.

Reviews from the transportation domain suggest the environment provides both cues and opportunities for people to engage in PA within their neighborhoods [5,6,58]. The current review supports such claims and provides evidence that these relationships exist across different populations. A plausible mechanism for observed associations is that neighborhood footpaths increase recreational and utilitarian walking/cycling by reducing the risk of falls (by providing people with opportunities to walk on a smooth, flat surfaces) and increasing perceptions of safety from traffic (due to barriers between roads and sidewalks) [59]. Sidewalk provision and town planning are under the control of various levels of government – local, state, federal. The various levels of government are capable of providing legislation that will promote or restrict community capacity to provide destinations (both utilitarian and recreational) and infrastructure (sidewalks and safe travel routes for non-motorized transportation) in neighborhood areas, influencing community behavior accordingly. The effects of such policy-level changes for changing PA in entire communities are potentially large and effective. Policy modifications are likely to influence PA by mandating the provision of safe environments close to the home (from both traffic and crime), useable green space and other recreational locations in which to engage in PA close to the home, active transport routes separate from vehicle traffic, and increases in school PE. Despite the difficulty of effecting change through policy or 'distal leverage points' [9], these and similar changes will likely contribute to longer lasting changes in behavior by making PA an easier "choice". Research examining how policy influences behavior [45,60,61] is promising, however research examining wider policy influences is needed to identify the most effective in creating environments producing sustainable increases in PA.

Because few studies have used objective measures of PA, the current review was limited to studies using measures of perceived environment. Although many of these studies used reliable measures of PA, such as the BRFSS [42,45], the lack of precise measurement of PA and environmental constructs may be obscuring true associations between PA and environmental characteristics [54]. Future studies are therefore encouraged to use both self-report and objective measures of PA [62], and when possible, combine self-report and objective measures of the environment to improve the predictive ability of studies. In addition, relatively few of included studies were conducted outside of the US, therefore research examining how environments influence activity in other countries is strongly encouraged.

Environmental changes may have differential effects on various sub-groups of the population (i.e. men and women may respond differently to similar aspects of the environment or environmental changes) [63]. The current review, limited to non-experimental studies, cannot answer these questions. As such, it is recommended that future research adopt a quasi-experimental approach to examine if similar environmental changes influence behaviors separately in regions where key socio-demographic measures are substantially different.

Some characteristics identified in this study, while likely difficult to alter, may be more readily manipulated than existing land densities and land-use mixes, which have received much attention within transportation literature [58]. Comparison studies demonstrate that rates of walking and cycling were higher in neighborhoods classified as transit orientated (higher connectivity) compared to neighborhoods classified as automobile orientated (lower connectivity) [64]. Additionally, modeling activities demonstrate that providing innovative block-cutting passages can increase connectivity of neighborhoods previously considered unfavorable to neighborhood walking [65]. Therefore, the provision of footpaths connecting previously unconnected neighborhoods and increasing connectivity may be a viable way to facilitate increased neighborhood PA. However, caution should be used in their planning and design to properly address safety and security concerns by providing good lighting and allowing residents and other street users' lines of sight into block-cutting passages. These modifications are effective strategies in reducing neighborhood crime [66,67].

The current research had several limitations that should be considered when examining the results. First, the sample was limited to studies that were published or accepted for publication, included adult populations, and used logistic regression analysis to determine associations between the perceived environment and activity. The limit to adult populations was imposed because very few studies examined features of the built or perceived environment in relation to the PA of children and youth. Thus, more research should be conducted with children and youth.

Second, two variables (PA facilities, presence of shops and services) displayed significant heterogeneity of variance suggesting that those groups of effect sizes do not represent a common population and that potential sources of heterogeneity should be sought. Unfortunately, the limited number of studies included in the analysis prohibited any search for moderators in the sample and thus the results for these two variables should be interpreted with caution. Likely sources of heterogeneity are the measurement instruments used to assess PA and the types of PA that were assessed in the original studies (e.g., walking, sufficient level of total PA). The possibility of PA measures and outcome measures as sources of heterogeneity should be examined in future analyses. Third, while several self-report measures with acceptable reliability [68,69] are available, studies used a variety of different measures to examine the perceived environment. Since alterations to measures may limit the possibility of making direct comparisons to previous studies, future research should use the most reliable measures in their entirety. Fourth, due to imprecise measurement of environmental constructs, variables were only included if a minimum of five ES were present. The number of ESs (n = 5) used in the analysis of outcome measures in previous meta-analyses [34,70] are similar to that used in the current study. However these studies used outcome measures (stroke, CVD) able to be measured with greater accuracy, and included larger within study sample sizes in pooled studies [34,70]. This minimum criterion may have reduced the number of constructs examined, however this delimitation was adopted to increase the confidence in ES estimation. Finally, all included studies were cross-sectional in design. Such designs may be subject to limitations including participants' self-selecting neighborhoods displaying design characteristics attractive to their own travel behaviors, attitudes [71], and PA preferences. For instance, using a pre-test/post-test study design, Krizek (2000) found that one-third of participants moved relatively short distances (<4 km) to neighborhoods possessing similar design characteristics, suggesting that neighborhood choice is based primarily on individual preference for a particular type of neighborhood [71]. Such limitations likely influence the results of included studies, and should be considered when interpreting the current results.

This quantitative review provides an objective summary of the association between a number of perceived environment variables and PA. The results should assist researchers in designing future research in the area. The small proportions of PA variance explained by the constructs reviewed suggest a need for the application of more comprehensive ecological models that include demographic, psychosocial, environmental, and biological variables.

## Competing interests

The author(s) declare that they have no competing interests.

## Authors' contributions

MD collated the literature, conducted the synthesis and meta-analysis and drafted the overall manuscript. JS assisted in the meta-analytic procedures and editing of the manuscript. KM conceived the study, and participated in its design and coordination. All authors read and approved the final manuscript.

**Table 2:** Crude and adjusted odds ratios of selected perceived environmental variables associations with physical activity.

Variable	Crude OR (95%CI)	Q	Adjusted^a ^OR (95%CI)	Q
**PA Facilities in Neighborhood**				
No	1.00		1.00	
Yes	0.99 (0.98–1.00)	Q(6) = 64.41*	1.20 (1.06–1.34)	Q(7) = 22.58*
				
**Sidewalks/Footpaths Present**				
No	1.00		1.00	
Yes	0.99 (0.97–1.01)	Q(6) = 8.47	1.29 (1.17–1.41)	Q(8) = 7.04
				
**Shops & Services in Walking Distance**				
No	1.00		1.00	
Yes	1.05 (0.80–1.29)	Q(3) = 3.81	1.30 (1.14–1.46)	Q(6) = 25.22*
				
**Heavy Traffic is Present**				
Yes	1.00		1.00	
No	1.00 (0.86–1.14)	Q(7) = 5.34	1.22 (1.08–1.37)	Q(6) = 6.32
				
**High Crime is Present**				
Yes	1.00		1.00	
No	0.90 (0.75–1.04)	Q(7) = 9.27	0.96 (0.80–1.11)	Q(4) = 5.89
				
**Street Lighting in Present**				
No	1.00		1.00	
Yes	0.94 (0.79–1.10)	Q(6) = 9.08	1.02 (0.90–1.15)	Q(7) = 5.39
				
**Unattended Dogs are a Problem**				
Yes	1.00		1.00	
No	0.90 (0.78–1.02)	Q(7) = 8.31	0.88 (0.75–1.02)	Q(4) = 2.30

^a^OR adjusted for those variables in original study

* Q is significant at the 0.05 level
